# Beet Pulp: An Alternative to Improve the Gut Health of Growing Pigs

**DOI:** 10.3390/ani10101860

**Published:** 2020-10-13

**Authors:** Hui Diao, Anran Jiao, Bing Yu, Jun He, Ping Zheng, Jie Yu, Yuheng Luo, Junqiu Luo, Xiangbing Mao, Daiwen Chen

**Affiliations:** 1 Key Laboratory for Animal Disease-Resistance Nutrition of China Ministry of Education, Institute of Animal Nutrition, Sichuan Agricultural University, Xinkang Road 46#, Ya’an 625014, China; diao_hui@hotmail.com (H.D.); harryjiaojiao@163.com (A.J.); ybingtian@163.com (B.Y.); hejun8067@163.com (J.H.); zpind05@163.com (P.Z.); jerryyujie@163.com (J.Y.); luoluo212@126.com (Y.L.); 13910@sicau.edu.cn (J.L.); 2 Animal Breeding and Genetics Key Laboratory of Sichuan Province, Sichuan Academy of Animal Science, No.7 Niusha Road, Chengdu 610066, China

**Keywords:** beet pulp, high-fiber diet, gut health, growing pigs

## Abstract

**Simple Summary:**

Improving disease resistance in pig is a major challenge faced by the pig industry. Intestinal health plays a critical role in modulating disease resistance in pigs. Dietary fiber has been widely recognized to prevent intestinal disorders in pigs. We found that feeding 5.74% crude fiber (obtained from beet pulp) to growing pigs could modulate gut microbiota composition and increase the short-chain fatty-acid content in the hindgut, suggesting that dietary supplementation with a high dose of fiber derived from beet pulp can help improve the gut health of growing pigs.

**Abstract:**

The present study aimed to investigate the effects of dietary fiber on the gut health of growing pigs. In total, 30 growing pigs with an initial average body weight of 45.8 ± 2.78 kg were divided into three groups with 10 replicates per treatment, and one pig per replicate. The treatments included a corn–soybean meal-based diet (control group, 1.5% crude fiber (CF)), corn–soybean meal + beet pulp-based diet (beet pulp group, 5.74% CF) and corn–soybean meal-based diet (feed intake-pairing group (pairing group); the feed intake was equal to the beet pulp group, 1.5% CF). The whole trial lasted 28 days. The beet pulp group had a longer length of the large intestine, higher weight of the small intestine and whole intestine, greater density of the large intestine and whole intestine, and higher villus height in the jejunum and ileum than the control group (*p* < 0.05). The messenger RNA (mRNA) expression levels of epidermal growth factor (EGF), glucagon-like peptide 2 (GLP-2), and glucagon-like peptide 2 receptor (GLP-2R) in the duodenum, EGF and GLP-2 in the jejunum, EGF in the ileum, and GLP-2 in the colon were higher in the beet pulp group than in the control group (*p* < 0.05). Moreover, the apparent total tract digestibility of crude ash, energy, dry matter (DM), and crude protein (CP) was lower in the beet pulp group than in the control group (*p* < 0.05), while the apparent total tract digestibility of CF, the activity of jejunal lactase, and the mRNA abundance of duodenal GLP-2 were higher in the beet pulp group than in the control and pairing groups (*p* < 0.05). In addition, the beet pulp group had more goblet cells in the colon, more *Bifidobacterium* spp. in the cecal digesta, higher concentrations of acetic acid and butyric acid in the cecal digesta, and higher mRNA abundance of duodenal regeneration protein Ⅲγ (REG-Ⅲγ), jejunal mucin 2 (MUC-2), and ileal G protein-coupled receptor 43 (GPR-43) than the control group (*p* < 0.05). However, these parameters did not differ between the control and pairing groups (*p* > 0.05). These findings indicate feeding a high-fiber diet (5.74% CF, obtained from beet pulp) to pigs could modulate the gut microbiota composition, increase the short-chain fatty-acid (SCFA) content in the hindgut, and improve gut health, which is independent of the feed intake.

## 1. Introduction

As one of the dietary nutrients affecting intestinal function, dietary fiber has been widely recognized to prevent intestinal disorders, such as post-weaning diarrhea and inflammatory bowel disease in pigs [1,2,3]. Hence, it can be considered a potential dietary constituent for alleviating stress-induced intestinal damage in pigs [4]. The beneficial effect of dietary fiber on the intestine is mainly attributed to the alteration of hindgut bacterial composition and the short-chain fatty-acid (SCFA) acetate, propionate, and butyrate contents, both of which have a potent anti-inflammatory effect [5]. Commensal bacteria and SCFA have been shown to modulate the intestinal barrier integrity. The messenger RNA (mRNA) expression levels of Occludin in CACO-2 cells have been found to be upregulated by *Lactobacillus* spp. in vitro [6]. *Escherichia coli* can disrupt tight junctions by dissociating the tight-junction proteins from epithelial cells [7]. A study using ovine mammary epithelial cells as an in vitro model revealed that propionic acid could inhibit the internalization of *Staphylococcus aureus* into the epithelial cells [8], while studies on CACO-2, IPEC-J2, and LS174T human colorectal cells demonstrated that butyrate could maintain intestine barrier function by increasing the expression of mucin and tight-junction protein genes and enhancing transepithelial electrical resistance [9,10,11]. These results suggest that bacteria and SCFA could affect the intestinal barrier integrity by regulating the expression levels of tight-junction proteins and mucin genes. Hence, dietary fiber may improve the function of the intestinal mucosa by modulating the intestinal bacteria and fermentation end-products in growing pigs.

Beet pulp has high levels of crude fiber (CF), nitrogen-free leachate, and crude protein (CP). Moreover, a large amount of l-arabinose polymer exists in the CF of beet pulp [12]. Pigs had a higher digestibility due to the fiber from beet pulp, mainly because of its high soluble fiber content [13]. Short-term studies using piglets revealed that piglets fed a diet containing beet pulp or wheat bran had a higher weight of the large intestine and fewer *Enterococcus* spp. in the feces [14,15]. The findings of these studies suggest that beet pulp has a positive effect on intestine microbiota composition. However, there is little available information on the systematic mechanism via which beet pulp promotes the intestinal health status. Consequently, we tested the hypothesis that dietary fiber obtained from beet pulp could affect gut health by regulating the intestinal morphology, nutrient digestion and absorption, and gut barrier function. In the present study, we modulated the dietary-fiber concentration by including beet pulp and systematically investigated the effects of a high-fiber diet on the gut health of growing pigs. Our findings will raise understanding about how dietary beet pulp inclusion beneficially influences the intestinal health in growing pigs.

## 2. Materials and Methods

### 2.1. Animals, Management, Diets, and Sample Collection

All of the experimental procedures and animal care were conducted on the basis of the Guide for the Care and Use of Laboratory Animals approved by the Institutional Animal Care Advisory Committee of Sichuan Agricultural University. All the animal protocols followed in the present study were admitted by the Animal Care and Use Committee of Sichuan Agricultural University under the permit number DKY-B20131704.

In total, 30 growing pigs (Duroc × Landrance × Yorkshire) with an initial average body weight of 45.8 ± 2.78 kg were randomly divided into three groups with 10 replicates per treatment and one pig per replicate. The treatments included a corn–soybean meal-based diet (control group, 1.5% CF), corn–soybean meal + beet pulp-based diet (beet pulp group, 5.74% CF), and corn–soybean meal-based diet (pairing group, the feed intake was equal to the beet pulp group, 1.5% CF). The whole trial lasted for 28 days. The experimental diet was prepared according to the nutrient recommendations of NRC (2012) required for pigs weighing 50–75 kg (Table 1).

The pigs were individually housed in metabolic cages (1.8 × 1.0 × 1.2 m) with woven wire flooring in an environmentally controlled room during the experimental period. All pigs were feed three times a day (8:00 a.m., 2:00 p.m., and 8:00 p.m.), with a little surplus in the trough as the criterion to judge satiety. The pigs in the control group and the beet pulp group were fed first every time. At the end of each feeding, the remaining feed was weighed, the feed intake was calculated in the beet pulp group, and an equal amount of feed was provided to the pairing group. The health status of each pig was checked at least once a day. All the pigs were afforded free access to water.

The fecal samples of each pig were acquired during days 24–27 and used to measure the apparent total tract digestibility (ATTD) of dry matter (DM), crude ash, CF, gross energy, ether extract (EE), and CP. On day 28, the blood samples were collected from the precaval vein into vacuum tubes and centrifuged for 10 min (3000× *g*, 4 °C).

All the pigs were anesthetized through intramuscular injection of 10 mg/kg body weight of zoletil 50 (Beijing PET Technology Co., LTD, Beijing, China) and then killed by exsanguination. After that, the abdomen was immediately opened, the lengths of both small and large intestine were scaled, and the samples of each part of the small intestine (duodenum, jejunum, and ileum) and colon were immediately separated and stored in 4% paraformaldehyde solution. Then, the ileal, cecal, and colonic digesta were collected. Following this, the weights of small and large intestines were measured. Finally, the duodenal, jejunal, ileal, and colonic mucosa were separated and immediately stored at −80 °C. The length, density, and weight of the intestine were calculated using previously described formulae [16]. Relative length of intestine (mm/g) = intestinal length/body weight. Relative density of intestine (g/cm) = intestinal weight/intestinal length. Relative weight of intestine (%) = intestinal weight/body weight.

### 2.2. Growth Performance

Individual pig body weight (BW) was recorded on day 0 and day 28 of the experimental period, and weighing was performed before pigs were fed. Feed consumption was recorded as the calculation of the amount of feed offered daily minus the remaining quantity in the feeder next morning on a pen basis during the experiment. These values were used to calculate average daily gain (ADG), average daily feed intake (ADFI), and ratio of feed to gain (F/G).

### 2.3. Histology of Intestine

Samples of pigs’ duodenum, jejunum, and ileum were collected, separated, and fixed in 4% paraformaldehyde solution. Following this, the samples were dehydrated and embedded in paraffin wax. Then, 5 μm sections of the intestinal samples were cut, installed, and stained with hematoxylin and eosin. Ten well-orientated sections were selected for each sample, and photographs were taken using a light microscope (Olympus, Tokyo, Japan) and a digital microscope camera (Olympus Optical Company, Guangzhou, China) at 100× magnification. Moreover, the number of goblet cells was counted by staining with periodic acid–Schiff and Alcian blue staining [17].

### 2.4. ATTD

All of the feed and fecal samples were assessed to measure the crude ash (method 942.05, AOAC, 1995), gross energy, CP (method 990.03, AOAC, 1995), CF (method 950.37, AOAC, 1995), EE (method 945.16, AOAC, 1995), and DM (method 930.15, AOAC, 1995) contents. The gross energy content was analyzed by bomb calorimetry (Parr Instrument 1563, Moline, IL, USA). ATTD was calculated using previously described formulae [18].

### 2.5. Enzyme Activities and Glucagon-Like Peptide 2 (GLP-2) Concentration

Supernatants of the jejunal mucosa samples were obtained according to the previously published procedures [19]. The jejunal mucosa was collected and homogenized with sterile saline (*w*/*v* = 1:9), after which the homogenates were centrifuged (1000× *g*, 4 °C, 15 min) and used for the analyses of total protein concentration, digestive enzyme activity, and GLP-2 concentration. The total protein concentrations of the jejunal mucosa samples were measured using the Bradford brilliant blue method. The lactase, sucrase, and maltase activities in the supernatants were assessed using an ultraviolet–visible light (UV–Vis) spectrophotometer (UV1100, Shanghai, China) with a kit produced by Nanjing Jiancheng Bioengineering Research Institute (Jiangsu, China). The GLP-2 concentration in the supernatants was measured using ELISA kits (R&D Systems, Minneapolis, MN, USA) and quantified using a microplate reader (BioTek Instruments, Winooski, VT, USA).

### 2.6. Real-Time Quantitative PCR

Total RNA was segregated from frozen duodenal, jejunal, ileal, and colonic mucosa using TRIzol reagent (Takara Bio Inc., Dalian, China) on the basis of the manufacturer’s protocols. The RNA samples were reverse-transcribed into complementary DNA (cDNA) with the PrimeScripteTM RT reagent kit (Takara Bio Inc., Dalian, China). Real-time PCR for the quantification of insulin-like growth factor 1 (IGF-1), GLP-2, epidermal growth factor (EGF), insulin-like growth factor 1 receptor (IGF-1R), glucagon-like peptide 2 receptor (GLP-2R), sodium/glucose cotransporter 1 (SGLT-1), glucose transporter type 2 (GLUT-2), solute carrier family 7 (SLC7A1), zinc transporters 1 (ZNT1), divalent metal transporter 1 (DMT1), mucin 1 (MUC1), regeneration protein Ⅲγ (REG-Ⅲγ), mucin 2 (MUC2), zonula occludens 1 (ZO-1), Occludin, G-protein-coupled receptor 41 (GPR41), and G-protein-coupled receptor 43 (GPR43) was performed using the CFX96 Real-Time PCR Detection System (Bio-Rad, Richmond, CA) on the basis of a previously published method [20]. In total, 10 μL quantitative fluorescent PCR reaction volume was used in this study, which consisted of 1 μL of cDNA, 0.5 μL of upstream and downstream primers, 5 μL of SYBR Premix Ex TaqTM, and 3 μL of RNase-free H_2_O. The PCR cycle conditions were as follows: 30 s at 95 °C, 10 s at 95 °C, and 25 s at 60 °C for a total of 40 cycles. Primer sequences are shown in Table S1 (Supplementary Materials). The primers were commercially synthesized by Invitrogen (Shanghai, China). The relative expression level of each gene in the tissues was calculated using β-actin as the reference gene.

### 2.7. Microbial Population Determination

The ileal, cecal, and colonic digesta were collected, and bacterial DNA was extracted with stool DNA kits (Omega Bio-tek, Doraville, CA, USA). The primers and fluorescent oligonucleotide probes (Table S2, Supplementary Materials) for total bacteria, *Eschericha coli*, *Lactobacillus* spp., *Bifidobacterium* spp., and *Bacillus* spp. were obtained according to previously published methods [21,22]. Quantitative real-time PCR was accomplished using the CFX96 Real-Time PCR Detection System (Bio-Rad, CA, USA). The PCR conditions and computing method were in accordance with those reported by Qi et al. (2011) [21].

### 2.8. SCFA

SCFAs in the frozen ileal and colonic digesta samples were isolated and quantified using the VARIAN CP-3800 gas chromatographic system (Palo Alto, CA, USA), as described previously [21]. In brief, the ileal and colonic digesta were collected and homogenized with distilled water (*w*/*v* = 1:1). The homogenates were centrifuged at 500× *g* for 10 min, after which 2 mL of the supernatant was collected and centrifuged at 12,000× *g* for 10 min. Next, 0.2 mL of 25% metaphosphoric acid was added to 1 mL of the supernatant, followed by standing for 30 min and centrifugation at 12,000× *g* for 10 min. The supernatant was then collected, and an equal volume of methanol was added and centrifuged at 12,000× *g* for 10 min. Finally, the supernatant was collected and stored at −20 °C for testing.

### 2.9. Statistical Analysis

All data were checked for normal distribution and homogeneity of variance using the Shapiro–Wilk and Levene’s tests, respectively, in SAS 8.2 (SAS Inst. Inc., NC, USA). When the data were recognized as normally distributed and exhibited homogeneity of variance, data were analyzed by one-way ANOVA and Duncan’s multiple comparison. During data analysis, each pig was considered as an experimental unit. A *p*-value < 0.05 was considered statistically significant. Results are displayed as the mean and standard error of the mean (SEM).

## 3. Results

### 3.1. Growth Performance

The impacts of dietary fiber on growth performance are shown in Table 2. From days 0 to 28, final weight (*p* = 0.001), ADFI (*p* < 0.001), and ADG (*p* < 0.001) decreased in the beet pulp and paring groups compared to the control group. No differences were observed in the three groups for the F/G during the overall period.

### 3.2. Intestinal Index

As shown in Table 3, the beet pulp group had a higher weight of the whole intestine (*p* = 0.012) and small intestine (*p* = 0.010), higher density of the whole intestine (*p* = 0.021) and large intestine (*p* = 0.006), and longer length of the large intestine (*p* = 0.025) than the control group. However, these parameters did not differ between the control and pairing groups (*p* > 0.05).

### 3.3. Intestinal Morphology

The beet pulp group had a higher villus height in the jejunum (*p* < 0.001) and ileum (*p* = 0.002), higher ratio of villus height-crypt depth in the duodenum (*p* < 0.001) and jejunum (*p* < 0.001) and more goblet cells in the ileum (*p* = 0.029) and colon (*p* = 0.028) than the control group (Table 4). The pairing group had a higher crypt depth in the duodenum (*p* = 0.002) and lower ratio of villus height–crypt depth in the duodenum (*p* < 0.001) and jejunum (*p* < 0.001) than the control group. The other parameters did not differ between the control and pairing groups (*p* > 0.05).

### 3.4. The Relative mRNA Expression Levels of Intestinal Development-Related Genes and GLP-2 Concentrations in the Jejunum

**Figure 1:**
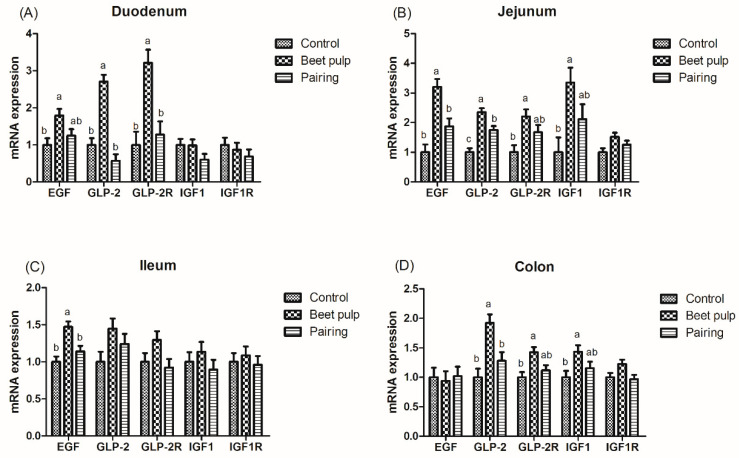
Effect of dietary fiber on the relative messenger RNA (mRNA) expression of intestinal development-related genes in growing pigs. Control, corn–soybean meal-based diet (1.5% CF). Beet pulp, corn–soybean meal + beet pulp-based diet (5.74% CF). Pairing, corn–soybean meal-based diet (the feed intake was equal to the beet pulp group, 1.5% CF). EGF, epidermal growth factor; IGF-1, insulin-like growth factor 1; GLP-2, glucagon-like peptide 2; IGF-1R, insulin-like growth factor 1 receptor; GLP-2R, glucagon-like peptide 2 receptor. ^a,b^ Within a row, means without a common superscript letter differ (*p* < 0.05).

The impacts of dietary fiber on the relative mRNA expression levels of intestinal development-related genes are shown in Figure 1. The beet pulp group had higher mRNA expression levels of EGF (*p* = 0.028), GLP-2 (*p* < 0.001), and GLP-2R (*p* = 0.002) in the duodenum, EGF (*p* = 0.001), GLP-2 (*p* < 0.001), GLP-2R (*p* = 0.017), and IGF-1 (*p* = 0.025) in the jejunum, EGF in the ileum (*p* = 0.003), and GLP-2 (*p* = 0.004), GLP-2R (*p* = 0.018), and IGF-1 (*p* = 0.049) in the colon than the control group. Moreover, the mRNA expression level of jejunal GLP-2 in the pairing group was higher than in the control group (*p* < 0.001). As shown in Table 5, the beet pulp group had a higher GLP-2 concentration in the jejunum than the control group (*p* = 0.041). The other parameters did not differ between the control and pairing groups (*p* > 0.05).

### 3.5. ATTD, Digestive Enzyme Activities, and mRNA Expression Levels of Digestion- and Absorption-Related Genes in the Small Intestine

**Figure 2:**
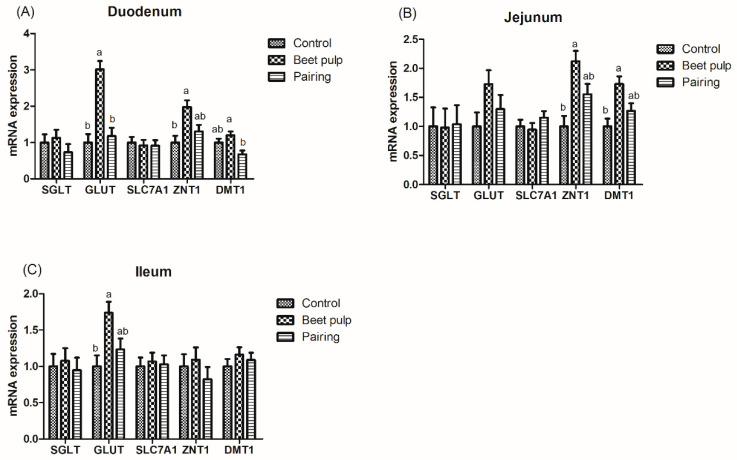
Effect of dietary fiber on the relative mRNA expression levels of intestinal digestion- and absorption-related genes in growing pigs. Control, corn–soybean meal-based diet (1.5% CF). Beet pulp, corn–soybean meal + beet pulp-based diet (5.74% CF). Pairing, corn–soybean meal-based diet (the feed intake was equal to the beet pulp group, 1.5% CF). SGLT-1, sodium/glucose cotransporter 1; GLUT-2, glucose transporter type 2; SLC7A1, solute carrier family 7; ZNT1, zinc transporters 1; DMT1, divalent metal transporter 1. ^a,b^ Within a row, means without a common superscript letter differ (*p* < 0.05).

As shown in Table 5, the ATTD of DM (*p* < 0.001), CP (*p* < 0.001), gross energy (*p* = 0.004), and crude ash (*p* < 0.001) was lower in the beet pulp group than in the control group, while the ATTD of CF was higher in the beet pulp group than in the control group (*p* < 0.001). In addition, as shown in Table 6, the activities of jejunal lactase (*p* < 0.001) and sucrase (*p* = 0.049) were higher in the beet pulp group than in the control group. Moreover, as shown in Figure 2, the beet pulp group had higher mRNA expression levels of GLUT-2 in the duodenum (*p* < 0.001) and ileum (*p* = 0.018), of ZNT1 in the duodenum (*p* = 0.011) and jejunum (*p* = 0.005), and of DMT1 in the jejunum (*p* = 0.009) than the control group. These parameters did not differ between the control and pairing groups (*p* > 0.05).

### 3.6. Intestinal Barrier Function

**Figure 3:**
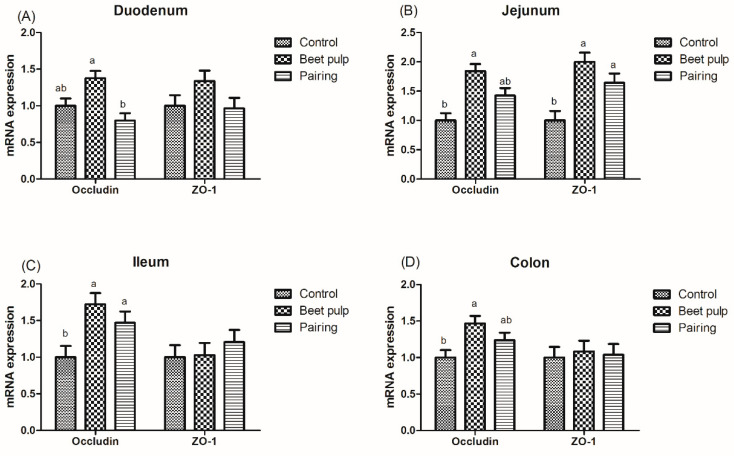
Effect of dietary fiber on the relative mRNA expression levels of intestinal tight-junction protein-related genes in growing pigs. Control, corn–soybean meal-based diet (1.5% CF). Beet pulp, corn–soybean meal + beet pulp-based diet (5.74% CF). Pairing, corn–soybean meal-based diet (the feed intake was equal to the beet pulp group, 1.5% CF). ZO-1, zonula occludens 1. ^a,b^ Within a row, means without a common superscript letter differ (*p* < 0.05).

**Figure 4:**
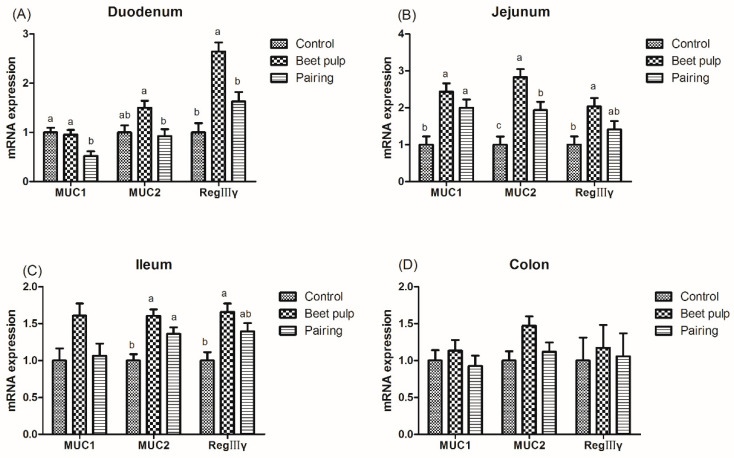
Effect of dietary fiber on the relative mRNA expression levels of intestinal mucin and REG-Ⅲγ in growing pigs. Control, corn–soybean meal-based diet (1.5% CF). Beet pulp, corn–soybean meal + beet pulp-based diet (5.74% CF). Pairing, corn–soybean meal-based diet (the feed intake was equal to the beet pulp group, 1.5% CF). MUC1, mucin 1. MUC2, mucin 2. REG-Ⅲγ, regeneration protein Ⅲγ. ^a,b^ Within a row, means without a common superscript letter differ (*p* < 0.05).

**Figure 5:**
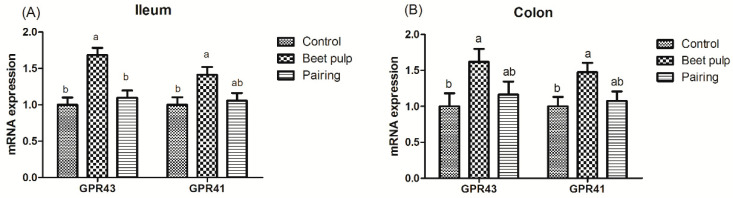
Effect of dietary fiber on the relative mRNA expression of intestinal short-chain fatty-acid (SCFA) receptors in growing pigs. Control, corn–soybean meal-based diet (1.5% CF). Beet pulp, corn–soybean meal + beet pulp-based diet (5.74% CF). Pairing, corn–soybean meal-based diet (the feed intake was equal to the beet pulp group, 1.5% CF). GPR41, G-protein-coupled receptor 41. GPR43, G-protein-coupled receptor 43. ^a,b^ Within a row, means without a common superscript letter differ (*p* < 0.05).

The beet pulp group had higher mRNA expression levels of ZO-1 (*p* = 0.004) and of occludin in the jejunum (*p* = 0.002), ileum (*p* = 0.002), and colon (*p* = 0.028) (Figure 3). The mRNA expression level of ileal ZO-1 in the pairing group was higher than that in the control group (*p* = 0.002). Moreover, pigs fed a high-fiber diet showed higher mRNA expression levels of REG-Ⅲγ in the duodenum (*p* < 0.001) and of MUC1, MUC2, and REG-Ⅲγ in the jejunum (*p* = 0.003, 0.001, and 0.026, respectively) and ileum (*p* = 0.049, 0.002, and 0.007, respectively) than those in the control group (Figure 4). The mRNA expression levels of jejunal (*p* = 0.001) and ileal (*p* = 0.002) MUC2 in the pairing group were higher than those in the control group, whereas the mRNA expression level of duodenal MUC1 (*p* = 0.009) in the pairing group was lower than that in the control group (*p* < 0.05). Moreover, the beet pulp group had more *Eschericha coli* in the colon (*p* = 0.041) and fewer *Bifidobacterium* spp. in the cecum (*p* = 0.026) than the control group (Table 7). In addition, pigs fed a high-fiber diet showed higher contents of propionic acid in the ileum (*p* = 0.033), butyric acid in the cecum (*p* = 0.048), and acetic acid (*p* < 0.001) and total SCFAs (*p* = 0.001) in the colon than those in the control group (Table 8). As shown in Figure 5, the mRNA expression levels of GPR41 and GPR43 in the ileum (*p* = 0.036 and 0.001, respectively) and colon (*p* = 0.047 and 0.049, respectively) in the pairing group were higher than those in the control group. The other parameters did not differ between the control and pairing groups (*p* > 0.05).

## 4. Discussion

As a monogastric species, pigs have limited ability to utilize dietary fiber [23]. Therefore, although pigs can tolerate a relatively high level of fiber, supplemental fiber in a pig’s diet is considered as an important factor affecting feed intake. Anguita et al. (2007) [24] and Zhang et al. (2013) [25] demonstrated that high fiber contents in diets can decrease the voluntary feed intake of the animals as a consequence of higher water retention capacity and gut fill, in agreement with the present study that a reduction in ADFI and ADG of pigs fed a beet pulp diet was found. However, the feed conversion rate of pigs was not affected by fiber levels, and an improved numerical value was even observed in beet pulp group, which indicated that feeding supplemental beet pulp possibly altered the intestinal digestive physiology and barrier function of pigs [26].

As an indigestible carbohydrate component of pig diet, fiber serves as the main fermentation substrate in the digestive tract, particularly for microorganisms in the hindgut, which maintains the normal physiological function of the digestive tract [27]. Intestinal weight can be affected by dietary fiber. Short-term studies using piglets revealed a higher relative weight of the large intestine in piglets fed a diet containing beet pulp [14,15]. Similar results were observed in growing pigs fed a diet containing 23% beet pulp (8.1% CF). The weight of the stomach and whole intestine significantly increased in these pigs [28]. In the present study, the beet pulp group had a higher weight of the whole intestine and small intestine and higher density of the large intestine than the control group, indicating that fiber could stimulate the growth of the intestinal mucosa and increase the mucosal weight [29]. It is well known that IGF-1, EGF, GLP-2, and its receptor (GLP-2R) are main regulators of intestine length [30,31]. Therefore, we further investigated the influences of beet pulp on the mRNA expression levels of intestinal development-related genes in the intestine. As expected, the mRNA expression levels of EGF, GLP-2, and GLP-2R in the duodenum, EGF and GLP-2 in the jejunum, EGF in the ileum, and GLP-2 in the colon were significantly enhanced by dietary beet pulp supplementation. In addition, increased GLP-2 concentration was observed in the present study, which further suggested the regulating ability of intestine development brought by beet pulp.

The intestinal morphology is also affected by dietary fiber. A high-fiber diet containing 10% wheat straw increased the proliferation rate of jejunal and ileal epithelial cells and increased the villus height and crypt depth in the jejunum and ileum in growing pigs [32]; these findings are consistent with those of the present study. Generally, the ratio of villus height to crypt depth represents the absorption capacity of the small intestine [33]. In the present study, villus height and the ratio of villus height to crypt depth in the jejunum were increased relative to those in the control group, which suggests that beet pulp influences pig intestinal absorption capacity by regulating morphological structures. However, in another study, no difference was noted in the intestinal morphology on 15% soybean husk, 15% oatmeal husk, or 20% alfalfa meal supplementation [34]. Chen et al. (2014) demonstrated that a diet supplemented with different sources of fiber (wheat fiber, pea fiber, soybean fiber, and corn fiber) caused variation in the structure of the small intestine in weaned piglets [35]. It appears, therefore, that, in addition to fiber levels, fiber sources may have a certain impact on the intestinal morphology.

Several studies have shown that dietary fiber levels and physicochemical properties affect nutrient digestion and absorption in pigs. A high-fiber diet containing 15% soybean husk, 15% oatmeal husk, or 20% alfalfa meal decreased the apparent digestibility of energy, DM, and nitrogen in growing pigs [34]. Moreover, a diet containing 20% beet pulp decreased the apparent digestibility of CP, crude fat, and organic matter, but increased the apparent digestibility of acidic detergent fiber and neutral detergent fiber in growing pigs [13]. In the present study, the beet pulp group had lower ATTD of DM, CP, gross energy, and crude ash and higher ATTD of CF than the control group, which shows that digestion ability of dietary fiber could be increased by beet pulp. Moreover, we probed the influences of beet pulp supplementation on intestinal enzymatic activity. Our results showed that the activities of jejunal lactase and sucrase in beet pulp group were higher than those in the control group, specifically, that the development of disaccharidase activities can be induced by beet pulp supplementation. Similarly, pigs fed a high-fiber diet stimulated the activities of maltose and lactase in the brush border of the intestinal mucosa, and elevated the activities of trypsin, chymotrypsin, and total hydrolytic protease in the duodenal digesta [15,36]. As previously mentioned, it is essential for providing sufficient lactase, maltase, and sucrase to degrade disaccharides [37]. In support of this notion, we speculated that beet pulp supplementation also can facilitate intestinal absorptive capacity through facilitating disaccharidase activities in growing pigs.

Fiber can affect the speed at which the digesta is passed through the intestine and reduce the retention time of the digesta in the small intestine, thus stimulating the compensatory secretion of endogenous digestive enzymes [38]. This led to a decrease in the nutrient digestibility and an increase in the activities of lactase and sucrase in pigs supplemented with beet pulp in the present study. Nonetheless, an inconsistent study found that a diet supplemented with 5% wheat bran or beet pulp had no significant effect on the nutrient digestibility in growing pigs [39]. Moreover, a diet supplemented with different sources of fiber had different digestibility both in weaned piglets and in growing pigs [40]. Taken together, these different results may be due to the effect of dietary fiber levels, physicochemical properties, and sources. In addition, higher mRNA expression levels of intestinal GLUT-2, ZNT1, and DMT1 were observed in the beet pulp group in our present. These findings are sufficient to suggest that beet pulp has certain effect on the intestinal digestion and absorption function.

A healthy and stable microbiota prevents the development of intestinal diseases and results in improved gut performance. Dietary fiber has been shown to selectively regulate gut bacteria, including stimulating the proliferation of probiotics and inhibiting the proliferation of potential pathogens [41,42,43,44]. Fiber depravation significantly altered the profile of the intestinal microbiota, greatly increased the number of mucus-degrading bacteria, and sharply decreased the number of fiber-decomposing bacteria in mice, thereby reducing the thickness of their mucus layer and increasing their susceptibility to pathogenic bacteria [45]. As a new dietary fiber, resistant starch can also promote the growth of beneficial bacteria, inhibit the number of harmful bacteria, promote the production of propionic acid and butyric acid in the colon [46], and stimulate the secretion of microbial enzymes [47]. Moreover, beet pulp can regulate the composition and quantity of intestinal microbiota. Previous studies demonstrated a lower number of *Enterococcus* in the feces of piglets fed a diet containing beet pulp [14,15]. Similarly, a diet containing 23% beet pulp significantly increased the numbers of *Lactobacillus* spp. and *Bifidobacterium* spp. and decreased the numbers of *Enterobacteriaceae* spp. and *Enterococcus* spp. in the hindgut of growing pigs [28]; this finding is consistent with the finding of the present study. With an increase in the *Bifidobacterium* spp. populations in the cecum of pigs fed the beet pulp diet, the establishment of *Escherichia coli* was inhibited possibly by a phenomenon known as colonization resistance [48]. Furthermore, our present study discovered that a diet containing 25% beet pulp increased the SCFA contents and SCFA receptor expression in the colon of pigs. It is well known that SCFAs are closely related to intestinal growth and barrier function. Hence, fiber may improve the intestinal health by modulating the microbiota composition and further changing the profile of SCFA [9,49]. In view of the previously mentioned reasoning, we summarized that the increased populations of *Bifidobacterium* spp. and the decreased populations of *Escherichia coli* in the intestine improved the gut health in response to dietary beet pulp supplementation, which may be associated with the changes in the intestinal barrier integrity.

The intestinal barrier integrity is mainly maintained by the tight junction, including the peripheral membrane protein ZO family and the transmembrane protein Occludin and Claudin families, which participate in the formation of the intestinal mucosal barrier and prevent antigens from invading the body [50]. In a study, measurement with Ussing-type chambers revealed that dietary fiber improved the intestinal function by alleviating the increase in intestinal cell permeability induced by 51Cr-EDTA [51]. An in vitro study revealed that the adherence of *Eschericha coli* to intestinal epithelial monolayers can give rise to the degradation of Occludin and ZO-1 and can downregulate the mRNA expression levels of both the genes, thereby diminishing the barrier function [52]. In our study, pigs in beet pulp group had lower counts of *Eschericha coli* and higher mRNA levels of ZO-1 and Occludin in intestine. These results suggest that the changed intestinal flora can influence the integrity of intestinal barrier by regulating the expression level of tight junction protein genes with beet pulp provided. As an important cell in the intestinal epithelium, goblet cells are found along the crypt–villus axis of the intestine and secreted mucins and peptide trifoliate factor, which contribute to the mucus layer in the intestine, providing an intestinal chemical barrier function [53]. A diet containing 23% beet pulp significantly increased the count of goblet cells in the jejunum of growing pigs [28]. Fiber intake increased the number of goblet cells in the intestinal mucosa compared to the fiber-free group, which is consistent with the results of the beet pulp group in this study [54]. In the current study, more goblet cells in the ileum and colon were noticed after beet pulp addition, accompanied by an upregulated MUC2 and REG-Ⅲγ transcriptional level in the intestine, indicating that fiber obtained from beet pulp also improved the intestinal chemical barrier function in growing pigs. Therefore, it can be speculated that fiber obtained from beet pulp can upregulate the expression of tight-junction proteins and antimicrobial proteins, thereby maintaining the intestinal barrier.

## 5. Conclusions

Feeding a high-fiber diet (5.74% CF, obtained from beet pulp) to pigs exerted beneficial effects by improving the intestinal health of growing pigs, which was independent of the feed intake. This behavior was closely related to the ameliorative intestinal morphology and enhanced absorption capacity, as well as integrated intestinal barrier function.

## Figures and Tables

**Table 1:** Ingredients and nutrient compositions of diets (air-dried basis %).

Ingredient	Control	Beet Pulp
Corn	84.65	57.62
Soybean meal	12.45	12.85
Soybean oil		2.19
Beet pulp		25.00
Dicalcium phosphate	0.98	1.18
Limestone	0.65	0.07
Choline chloride	0.10	0.10
NaCl	0.30	0.30
Vitamin complex ^1^	0.03	0.03
Lys (78%)	0.45	0.28
*dl*-Met (99%)	0.02	0.05
Thr (98.5%)	0.05	0.01
Trp (98.5%)	0.03	0.02
Mineral complex ^2^	0.30	0.30
Total	100.00	100.00
Calculated composition		
DE, Mcal/kg	13.72	13.72
Crude protein	12.72	12.71
Crude fiber	1.50	5.74
Calcium	0.60	0.60
Total phosphorus	0.53	0.53
Lys	0.86	0.85
Met	0.49	0.49
Thr	0.53	0.52
Trp	0.15	0.15
Analyzed composition		
Crude protein	13.41	13.40
Crude ash	3.27	3.65
Crude fiber	2.68	6.71
Dry matter	89.84	90.23
Total energy, MJ/kg	3959.23	4077.08
Ether extract	4.21	4.69

^1^ The premix provided the following per kg diet: Vitamin A, 3307 IU; Vitamin D_3_, 1350 IU; Vitamin E, 14.4 mg; Vitamin K_3_, 1.8 mg; Vitamin B_2_, 3.6 mg; Vitamin B_6_, 1.8 mg; Vitamin B_12_, 14.4 μg; folic acid, 0.72 mg; nicotinic acid, 8.4 mg; biotin, 90 μg; *d*-pantothenic acid, 9 mg. ^2^ The premix provided the following per kg diet: Fe, 50 mg; Cu, 3.5 mg; Mn, 2 mg; Zn, 50 mg; I, 0.14 mg; Se, 0.15 mg.

**Table 2:** Effect of dietary fiber on growth performance in growing pigs.

Items	Control	Beet Pulp	Pairing	SEM	*p*-Values
Initial BW	45.722	45.778	45.833	0.478	0.987
28 d BW	75.944 ^a^	68.667 ^b^	68.167 ^b^	1.114	0.001
ADFI (kg)	2.785 ^a^	1.964 ^b^	1.964 ^b^	0.043	<0.001
ADG (kg)	1.079 ^a^	0.815 ^b^	0.800 ^b^	0.044	<0.001
F/G	2.587	2.462	2.573	0.119	0.725

Control, corn–soybean meal-based diet (1.5% CF). Beet pulp, corn–soybean meal + beet pulp-based diet (5.74% CF). Pairing, corn–soybean meal-based diet (the feed intake was equal to the beet pulp group, 1.5% CF). BW, body weight; ADFI, average daily feed intake; ADG, average daily gain; F/G, ratio of feed to gain; SEM, standard error of the mean. ^a,b^ Within a row, means without a common superscript letter differ (*p* < 0.05).

**Table 3:** Effect of dietary fiber on intestinal index in growing pigs.

Items	Control	Beet Pulp	Pairing	SEM	*p*-Values
Length of SI, cm/g	2.237	2.245	2.421	0.095	0.345
Length of LI, cm/g	0.581 ^b^	0.718 ^a^	0.625 ^ab^	0.030	0.025
Length of I, cm/g	2.819	2.963	3.045	0.112	0.383
Density of SI, g/cm	2.183	2.598	2.452	0.113	0.073
Density of LI, g/cm	1.729^b^	2.105 ^a^	1.658 ^b^	0.079	0.006
Density of I, g/cm	3.912 ^b^	4.702 ^a^	4.110 ^ab^	0.171	0.021
Weight of SI, %	0.976 ^b^	1.164 ^a^	1.013 ^b^	0.036	0.010
Weight of LI, %	2.984	2.939	2.679	0.115	0.178
Weight of I, %	1.388 ^b^	1.596 ^a^	1.352 ^b^	0.050	0.012

Control, corn–soybean meal–based diet (1.5% CF). Beet pulp, corn–soybean meal + beet pulp-based diet (5.74% CF). Pairing, corn–soybean meal-based diet (the feed intake was equal to the beet pulp group, 1.5% CF). SI, small intestine; LI, large intestine; I, intestine. ^a,b^ Within a row, means without a common superscript letter differ (*p* < 0.05).

**Table 4:** Effect of dietary fiber on intestinal morphology and goblet cells in growing pigs.

Items	Control	Beet Pulp	Pairing	SEM	*p*-Values
Duodenum					
Villus height, μm	830.540	886.010	838.840	24.809	0.279
Crypt depth, μm	494.610 ^b^	438.580 ^b^	577.910 ^a^	19.861	0.002
Villus height–crypt depth	1.704 ^b^	2.040 ^a^	1.459 ^c^	0.058	<0.001
Jejunum					
Villus height, μm	672.270 ^b^	836.460 ^a^	639.020 ^b^	14.975	<0.001
Crypt depth, μm	366.460	395.300	425.060	16.778	0.093
Villus height: crypt depth	1.864 ^b^	2.129 ^a^	1.512 ^c^	0.060	<0.001
Ileum					
Villus height, μm	406.679 ^b^	446.195 ^a^	404.529 ^b^	6.849	0.002
Crypt depth, μm	366.680	355.040	353.240	16.240	0.821
Villus height–crypt depth	1.117	1.262	1.171	0.047	0.137
Goblet cells					
Ileum	142.150 ^b^	194.440 ^a^	162.080 ^ab^	11.589	0.029
Colon	179.170 ^b^	212.330 ^a^	179.290 ^b^	8.354	0.028

Control, corn–soybean meal-based diet (1.5% CF). Beet pulp, corn–soybean meal + beet pulp-based diet (5.74% CF). Pairing, corn–soybean meal-based diet (the feed intake was equal to the beet pulp group, 1.5% CF). ^a,b^ Within a row, means without a common superscript letter differ (*p* < 0.05).

**Table 5:** Effect of dietary fiber on apparent total tract digestibility in growing pigs, %.

Items	Control	Beet Pulp	Pairing	SEM	*p*-Values
EE	82.032 ^ab^	80.560 ^b^	84.368 ^a^	0.600	0.004
DM	92.680 ^a^	87.382 ^b^	93.150 ^a^	0.476	<0.001
Crude ash	77.220 ^a^	62.843^b^	76.628 ^a^	1.326	<0.001
CP	90.677 ^a^	80.060 ^b^	90.950 ^a^	0.846	<0.001
CF	72.173 ^b^	81.413 ^a^	75.553 ^b^	0.939	<0.001
Energy	92.894 ^a^	88.612 ^b^	93.437 ^a^	0.350	<0.001

Control, corn–soybean meal-based diet (1.5% CF). Beet pulp, corn–soybean meal + beet pulp-based diet (5.74% CF). Pairing, corn–soybean meal-based diet (the feed intake was equal to the beet pulp group, 1.5% CF). EE, ether extract; DM, dry matter; CP, crude protein; CF, crude fiber. ^a,b^ Within a row, means without a common superscript letter differ (*p* < 0.05).

**Table 6:** Effect of dietary fiber on digestive enzyme activities and glucagon-like peptide 2 (GLP-2) concentration in jejunum of growing pigs.

Items	Control	Beet Pulp	Pairing	SEM	*p*-Values
Total protein, mg prot/g	56.071	54.004	59.583	3.959	0.622
Lactase, U/mg prot	15.161 ^b^	30.125 ^a^	18.360 ^b^	1.332	<0.001
Sucrase, U/mg prot	75.273 ^b^	110.209 ^a^	84.824 ^ab^	7.192	0.049
Maltase, U/mg prot	428.315	514.422	449.275	26.515	0.104
GLP-2, pmol/g prot	1.680 ^b^	2.143 ^a^	1.655 ^ab^	0.090	0.041

Control, corn–soybean meal-based diet (1.5% CF). Beet pulp, corn–soybean meal + beet pulp-based diet (5.74% CF). Pairing, corn–soybean meal-based diet (the feed intake was equal to the beet pulp group, 1.5% CF). GLP-2, glucagon-like peptide 2. ^a,b^ Within a row, means without a common superscript letter differ (*p* < 0.05).

**Table 7:** Effect of dietary fiber on the numbers of *Eschericha coli*, *Lactobacilli* spp., *Bifidobacterium* spp., and *Bacillus* spp. in digesta of ileum, cecum, and colon in growing pigs (log copies/g).

Items	Control	Beet Pulp	Pairing	SEM	*p*-Values
Ileum					
Total bacteria	10.072	9.708	9.763	0.117	0.107
*Bacillus* spp.	10.370	10.420	10.383	0.043	0.708
*Lactobacillus* spp.	7.373	7.552	7.413	0.128	0.602
*Eschericha coli*	8.473 ^ab^	7.813 ^b^	8.790 ^a^	0.211	0.024
*Bifidobacterium* spp.	8.763	9.325	8.832	0.249	0.267
Cecum					
Total bacteria	11.423 ^a^	11.072 ^b^	11.350 ^a^	0.057	0.004
*Bacillus* spp.	10.473	10.488	10.492	0.029	0.895
*Lactobacillus* spp.	8.322	8.260	8.253	0.100	0.870
*Eschericha coli*	8.835 ^ab^	8.465 ^b^	9.277 ^a^	0.161	0.016
*Bifidobacterium* spp.	9.352 ^b^	9.703 ^a^	9.358 ^b^	0.087	0.026
Colon					
Total bacteria	11.580	11.477	11.482	0.063	0.453
*Bacillus* spp.	10.708	10.708	10.667	0.028	0.493
*Lactobacillus* spp.	8.218	8.520	8.273	0.165	0.420
*Eschericha coli*	8.997 ^a^	8.622 ^b^	8.820 ^ab^	0.057	0.041
*Bifidobacterium* spp.	9.592	9.137	9.410	0.151	0.151

Control, corn–soybean meal-based diet (1.5% CF). Beet pulp, corn–soybean meal + beet pulp-based diet (5.74% CF). Pairing, corn–soybean meal-based diet (the feed intake was equal to the beet pulp group, 1.5% CF). ^a,b^ Within a row, means without a common superscript letter differ (*p* < 0.05).

**Table 8:** Effect of dietary fiber on the SCFA concentrations in digesta of ileum, cecum, and colon in growing pigs, μmol/g.

Items	Control	Beet Pulp	Pairing	SEM	*p*-Values
Ileum					
Acetic acid	17.854	22.371	16.681	1.531	0.058
Propionic acid	0.444 ^b^	0.917 ^a^	0.613 ^ab^	0.108	0.033
Butyric acid	0.944	1.288	0.968	0.114	0.107
Total SCFA	19.242 ^ab^	24.576 ^a^	18.262 ^b^	1.615	0.042
Cecum					
Acetic acid	50.575 ^b^	67.167 ^a^	54.377 ^b^	2.385	0.002
Propionic acid	22.190	25.219	22.120	2.232	0.553
Butyric acid	6.407 ^b^	7.661 ^a^	6.410 ^b^	0.297	0.048
Total SCFA	79.172 ^b^	100.047 ^a^	82.906 ^ab^	3.743	0.006
Colon					
Acetic acid	42.251 ^b^	61.839 ^a^	40.079 ^b^	2.498	<0.001
Propionic acid	17.161	18.617	15.408	1.435	0.327
Butyric acid	10.563 ^ab^	12.554 ^a^	9.783 ^b^	0.654	0.035
Total SCFA	69.975 ^b^	93.010 ^a^	65.269 ^b^	3.869	0.001

Control, corn–soybean meal-based diet (1.5% CF). Beet pulp, corn–soybean meal + beet pulp-based diet (5.74% CF). Pairing, corn–soybean meal-based diet (the feed intake was equal to the beet pulp group, 1.5% CF). SCFA, short-chain fatty acids. ^a,b^ Within a row, means without a common superscript letter differ (*p* < 0.05).

## Supplementary Materials

The following are available online at https://www.mdpi.com/2076-2615/10/10/1860/s1, Table S1: Primer sequences and annealing temperature, Table S2: Primers and probes for real-time PCR analysis of bacteria.
