# Supplementary material for: Beet Pulp: An Alternative to Improve the Gut Health of Growing Pigs
Source: Animals (Basel). 2020 Oct 13;10(10):1860. doi: 10.3390/ani10101860 (PMC7600662; doi:10.3390/ani10101860)
Supplement: Supplementary file 1 [file animals-10-01860-s001.pdf]

animals

MDPI

Table S1 Primer sequences and annealing temperature

Target gene	Forward primer 5′-3′	Reverse primer 5′-3′	Product length	Accession number
EGF	ATCTCAGGAATGGGAGTCAACC	TCACTGGAGGATGGAATACAGC	165	NM_214020.1
GLP-2	ACTCACAGGGCACGTTTACCA	AGGTCCCTTCAGCATGTCTCT	149	NM_005671883.1
GLP2R	GACCCTCTCTTGTGTCTTCGTA	AAGATGACGTCCTTCGCCAG	120	NM_001246266.1
IGF-1	CTGAGGAGGCTGGAGATGTACT	CCTGAACTCCCTCTACTTGTGTTC	137	NM_001097417.1
IGF-1R	TTCGCCAGATCCTAGGGGAG	TCCCAGCTTTGATGGTCAGG	120	NM_214172.1
SGLT-1	GCAACAGCAAAGAGGAGCGTAT	GCCACAAAACAGGTCATAGGTC	137	NM_001164021.1
GLUT-2	GACACGTTTTGGGTGTTCCG	GAGGCTAGCAGATGCCGTAG	149	NM_001097417.1
SLC7A1	TCTTTGCAGGTCGTTTGGGA	GGCTGATCACCTGTTGGAGT	137	NM_001012613.1
DMT1	GCAGGTGGTTGACGTCTGTA	CACGCCCCCTTTGTAGATGT	100	NM_001128440.1
ZnT1	TGCTCTGCATGCTGTTACTGA	TGGAAGGAGTCCGAGAGCAT	97	NM_001139470.1
Occludin	CAGGTGCACCCTCCAGATTG	GGACTTTCAAGAGGCCTGGAT	110	NM_001163647.2
ZO-1	CTGAGGGAATTGGGCAGGAA	TCACCAAAGGACTCAGCAGG	105	XM_013993251.1
REG3γ	GGCTTGGAACCAAATGCTGG	TAGCCAGGGTATGAGCTGGT	101	XM_005662419.1
MUC1	GTGCCGCTGCCCACAACCTG	AGCCGGGTACCCCAGACCCA	141	XM_001926883.5
MUC2	GGTCATGCTGGAGCTGGACAGT	TGCCTCCTCGGGGTCGTCAC	181	XM_013989745.1
β-actin	TCTGGCACCACACCTTCT	TGATCTGGGTCATCTTCTCAC	114	DQ178122

EGF, epidermal growth factor. IGF-1, insulin-like growth factor-1. GLP-2, glucagon-like peptide-2. IGF-1R, insulin-like growth factor-1 receptor. GLP-2R, glucagon-like peptide-2 receptor. SGLT-1, sodium/glucose cotransporter 1. GLUT-2, glucose transporter type 2. SLC7A1, solute carrier family 7. ZNT1, zinc transporters-1. DMT1, divalent metal transporter-1. ZO-1, zonula occludens. MUC1, mucin 1. MUC2, mucin 2. REGⅢγ, regeneration protein Ⅲγ. GPR41, G protein coupled receptor-41. GPR43, G protein coupled receptor-43.

Table S2 Primes and probes for real-time PCR analysis of bacteria.

Items	Primer/probe name and sequence(5'-3')	Product length/bp
Escherichia coli	DC-F,CATGCCGCGTGTATGAAGAA	
	DC-R,CGGGTAACGTCAATGAGCAAA	96
	DC-P,(FMA)AGGTATTAACTTTACTCCCTTCCTC(BHQ-1)	
Lactobacillus	RS-F,GAGGCAGCAGTAGGGAATCTTC	
	RS-R,CAACAGTTACTCTGACACCCGTTCTTC	126
	RS-P,(FMA)AAGAAGGGTTTCGGCTCGTAAAACTCTGTT(BHQ-1)	
Bifidobacterium	SQ-F,CGCGTCCGGTGTGAAAG	
	SQ-R,CTTCCCGATATCTACACATTCCA	121
	SQ-P, (FMA) ATTCCACCGTTACACCGGGAA(BHQ-1)	
Bacillus	YB-F,GCAACGAGCGCAACCCTTGA	
	YB-R,TCATCCCCACCTTCCTCCGGT	92
	YB-P, (FMA)CGGTTTGTCACCGGCAGTCACCT(BHQ-1)	
Total bacteria	Eub338F,ACTCCTACGGGAGGCAGCAG	
	Eub518R,ATTACCGCGGCTGCTGG	200
